# Interventions to reduce inequalities for pregnant women living with disadvantage in high-income countries: an umbrella review

**DOI:** 10.1186/s12889-025-22283-5

**Published:** 2025-03-25

**Authors:** Nicola Vousden, Dorothea Geddes-Barton, Stephanie J Hanley, Nia Roberts, Marian Knight

**Affiliations:** 1 Nuffield Department of Population Health, National Perinatal Epidemiology Unit, University of Oxford, Old Road Campus, Headington, Oxford OX3 7LF UK; 2 Department of Applied Health Sciences, College of Medicine and Health, University of Birmingham, Edgbaston, Birmingham, B15 2TT UK; 3 Bodleian Health Care Libraries, University of Oxford, Old Road Campus, Headington, Oxford, OX3 7LF UK

**Keywords:** Pregnancy, Inequalities, Disadvantage, Deprivation, Social risk, Umbrella review, Effectiveness

## Abstract

**Background:**

Women facing multiple disadvantage such as financial poverty, poor mental health or domestic abuse, may experience inequalities in health prior to and during pregnancy, as well as into early motherhood. This can have lifelong intergenerational impacts. The primary aim of this overview was to identify the breadth and efficacy of interventions that work across health and social care to reduce inequalities in maternal or child health. The second aim was to explore their relevance to women with lived experience.

**Methods:**

An overview of systematic reviews and meta-analyses from high-income countries that aim to reduce inequalities for women with social disadvantage during pregnancy was performed. Searches were conducted in eight electronic databases up to August 2023 and supplemented with grey literature searches. We included any individual, hospital, or community level activities specific to women during the pre-conception, antenatal or postpartum period up to one year after. The protocol was registered. Two workshops with women with lived experience of disadvantage explored the relevance of identified interventions, and gaps in evidence, in relation to their experiences.

**Results:**

A total of 36 reviews, including 734 primary studies, were included in the narrative synthesis. The majority of reviews included studies undertaken in North America and were of critically low or low quality. Interventions were grouped into 11 categories. The majority of interventions were aimed at single social exposures and targeted individual behavior during pregnancy and the postnatal period. Some at risk populations were excluded from all reviews. There was potential benefit of home-based interventions, psychosocial interventions, models of maternity care and interdisciplinary programs of care for some population groups, across a range of maternal and child health outcomes. Our lived experience group felt these interventions had potential to meet their shared needs for advocacy, support and information, but they should also consider culture, past trauma and factors underpinning pregnancy such as housing and finances.

**Conclusions:**

Further high-quality research is required to demonstrate efficacy and cost-effectiveness of potentially effective interventions in the European health systems. Additional research gaps include interventions prior to pregnancy, culture informed care and upstream determinants of health (PROSPERO: CRD42023455502).

**Supplementary Information:**

The online version contains supplementary material available at 10.1186/s12889-025-22283-5.

## Background

Nearly one in ten women who die in pregnancy and the postnatal period in the United Kingdom are reported to experience severe and multiple disadvantage [1]. The true value is likely higher as the most common underlying disadvantages, mental health diagnosis, substance use and domestic abuse, are often poorly recorded [1]. The increased risk to maternal health associated with social adversity has been identified to be approximately equivalent to common medical comorbidities [2]. Psychosocial risk factors recorded prior to, or during, early pregnancy are also associated with increased risks for the infant, including being born low birthweight (LBW) or preterm (PTB), or experiencing injury and death during the year after birth [3].

Current guidelines from the UK National Institute for Health and Care Excellence recommend that women with “complex social factors” should be identified so additional support may be provided. This guidance, written in 2010, focuses on four key areas, women with alcohol or drug misuse, domestic abuse, recent migrant or asylum seeker status and young mothers [4]. However, the guidance relied heavily on expert opinion due to the lack of high-quality research. Subsequent reviews of this guidance recommended that a wider range of social complexity were included such as mental health and homelessness [5]. In addition, women often do not experience these factors in isolation. Multiple exposures may be present, accumulate and interact over time, to have a greater impact than their sum [6]. It is vital therefore that interventions aim to address multiple complex needs, rather than focus on discrete populations with a single risk factor or exposure.

Existing reviews have explored interventions to reduce inequalities focusing on model or place of antenatal care [7, 8] or short-term outcomes of pregnancy such as preterm birth [9]. However, the impacts of socioeconomic disadvantage cannot be prevented by maternity alone. There is a need to identify and understand interventions with a -multi-disciplinary approach, that can impact prior to pregnancy to improve pregnancy outcomes, as well as in the postnatal period to improve maternal and infant outcomes [10, 11]. Therefore, this systematic review aimed to synthesise the quantitative literature in order to identify what interventions exist, and how effective they are at reducing inequalities in maternal and child health for pregnant women living with disadvantage in high-income countries.

## Methods

### Registration and protocol adherence

This review was registered on 22/08/2023 (PROSPERO, no: CRD42023455502) and presented according to the Preferred Reporting Items for Overviews of Systematic Reviews (Supplementary Table S1). The methods are described in more detail in the protocol [12]. The eligibility criteria is described in Table 1.

**Table 1:** Eligibility criteria and population, intervention, comparison and outcome (PICO) framework

**Population**	One or more of the following exposures prior to or during pregnancy: substance or alcohol misuse (excluding tobacco), involvement in the judicial system / prison, victim of modern slavery, homelessness or insecure housing, socioeconomic deprivation (measured for individual), domestic abuse or intimate partner violence, experience of sex work, underserved migrant women, and women from minoritised ethnic groups including Gypsy, Roma and Travelling communities [13]. We intended to include young mothers age < 20 but a number of relevant reviews had defined young mothers as < 24 so this age category was extended. We intended to include all reviews for women with a mental health diagnosis but subsequently identified an umbrella review of interventions to prevent perinatal depression [14] and two umbrella reviews of psychological [15] and complementary therapies [16] to treat depression in pregnancy therefore reviews of women with a mental health diagnosis were only included if there was either an additional form of disadvantage or different intervention
Interventions	Any individual, hospital or community level activity specific to women during the pre-conception, antenatal or postpartum period up to one year after birth
Control	Any comparison or control group
Outcome	All quantitative outcomes related to inequalities in maternal and child health up to five years of age are reported

#### Inclusion and exclusion criteria

We included reviews and meta-analyses as defined by the Cochrane Collaboration’s Handbook definition of systematic reviews of interventions, undertaken in high-income countries as defined by the World Bank in 2024, published in any language from 1st January 2013 to 17th August 2023. This 10-year period was selected as per the Joanna Briggs Institute guidance as they are considered to represent the contemporaneous evidence base over the previous 30 years [17].

#### Data collection and appraisal

We searched 8 databases: Medline(OvidSP)[1946-present], Embase(OvidSP)[1974-present], PsycINFO(OvidSP)[1806-present], CINAHL(EBSCOHost)[1982-present], ASSIA(Proquest)[1985-present], Science Citation Index and Social Science Citation Index(Web of Science Core Collection)[1900-present]. Systematic review repositories: Cochrane Database of Systematic Reviews, Database of Abstracts of Reviews of Effects were also searched. (Supplementary Table S2). The search strategy was comprised of a combination of title, abstract, author keywords and subject headings for 4 PICO concepts. In built filters were used to limit the search to systematic reviews or meta-analyses. Protocols, conference abstracts, dissertations and pre 2013 papers were excluded prior to import to Covidence. Backward and forward citation searches and an extensive grey literature search were undertaken.

Retrieved titles and abstracts were independently assessed by two reviewers against the inclusion/exclusion criteria. Relevant full texts were reviewed independently by two reviewers without disagreement. Data extraction for the first 20% of studies was undertaken independently by two authors (NV, DGB, SH), with good agreement (93.8%) so the remainder were extracted by a single author (NV). There were no major discrepancies.

The AMSTAR 2 appraisal tool was used to assess quality of included reviews [18]. As planned, two authors assessed the quality of each text for 20% of studies (NV, DGB, SH) with minor discrepancies resolved by consensus (13.3%). There were no major discrepancies. The remainder were assessed by a single author (NV).

### Data synthesis

Each review was categorized by its primary population as identified in the aim, timing of intervention and maternal and child health outcomes. A narrative synthesis was undertaken as planned due to expected heterogeneity. Overlap of the primary studies within systematic reviews was assessed using a pairwise intersection heat map [19]. As described in our protocol, where a meta-analysis or sub-analyses met our inclusion criteria we present these meta-analysis results. Where relevant reviews included at least three primary studies that met our inclusion criteria, we included them and narratively resynthesized the outcome data. Where a review of multiple intervention types had only one primary study describing an intervention, the paper is described but results not presented.

### Lived experience input

We held worked with women with recent lived experience of multiple social disadvantage in pregnancy to contextualise the results of this review (Panel 1). We explored how their social circumstances influenced their experience of care during and after pregnancy, and whether the identified interventions were relevant to their experiences. We identified gaps in the evidence base where interventions might have improved their experience.

## Results

### Selected studies

**Fig. 1:**
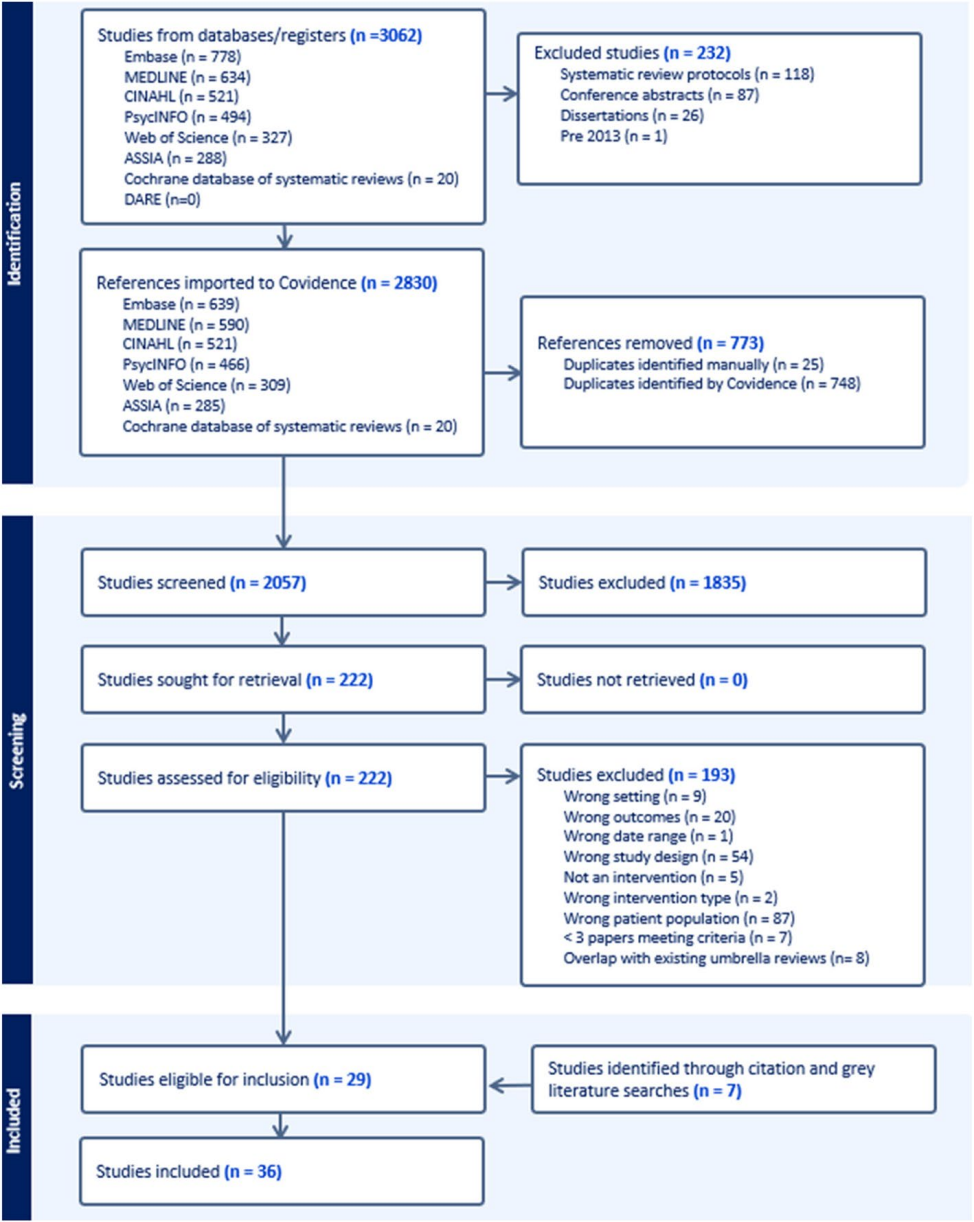
Flow diagram

From 2057 studies after duplicate removal, we excluded 1835 studies at the screening and 192 at the full-text stage, resulting in 29 publications included. Citation and grey literature searching identified a further seven publications (Fig. 1). Of the 11 meta-analyses, seven were of randomised controlled trials (RCT) and four were of both RCT and other comparative studies (Table 2). A total of 734 individual papers were included in the 36 reviews, 468 of these, including data on more than 435,000 women, met our criteria for inclusion and their findings are reported.

**Table 2:** Characteristics of included systematic reviews

Characteristic	Reviews included in overview *n*(%)	References for reviews in each category
Type of review		
- Narrative	25 (69.4)	[20–30]
- Meta-analysis	11 (30.5)	[7, 13, 31–53]
At least one primary study undertaken in:		
- UK	8 (22.2)	^a^
- USA or Canada	34 (94.4)	^a^
- Australia	9 (25)	^a^
- Other European Country	8 (22.2)	^a^
- South Asia	6 (16.7)	^a^
- Southern America	2 (5.6)	^a^
Primary source of disadvantage identified as exposure:		
- Young mothers	6 (16.7)	[20, 21, 35, 41, 48, 54]
- Minoritised ethnic groups	10 (27.3)	[13, 29, 36, 37, 42, 45, 46, 49, 50, 53]
- Intimate Personal Violence	4 (11.1)	[24, 31, 39, 40]
- Mental Health	7 (3 as dual exposure^b^) (19.4)	[22, 26, 27, 35–38]
- Socioeconomic disadvantage	11 (30.5)	[7, 23, 28, 30, 32–34, 43, 44, 51, 52]
- Substance misuse	1 (2.8)	[25]
- Criminal Justice System	0 (0.0)	
- Victims of modern slavery	0 (0.0)	
- Homelessness or insecure housing	0 (0.0)	
- Experience of sex work	0 (0.0)	
Timing of Intervention		
- Pre-conception	0 (0.0)	
- Pregnancy	12 (33.3)	[7, 20, 22, 25, 28, 30, 34, 39, 43, 44, 46, 48]
- Postnatal	3 (8.3)	[21, 27, 31]
- Pregnancy & postnatal	21 (58.3)	^a^
Outcome domain		
- Maternal Health e.g., depression, interpersonal violence, or unintended repeat pregnancy	24 (66.6)	^a^
- Breastfeeding	13 (36.1)	^a^
- Infant / Child Health e.g., preterm birth, low birth weight	21 (7)	^a^
- Attendance or engagement with care e.g., timing of the first antenatal appointment or uptake of childhood immunizations or family planning	10 (27.8)	^a^

^a^In Supplementary table S3

^b^Three reviews had dual exposures such as young mothers or minoritized ethnic groups *with* a mental health diagnosis

The characteristics of all included reviews are summarized in Table S3. The vast majority of reviews included at least one study from North America (*n* = 34, 94.4%) and interventions delivered during both pregnancy and the postnatal period (*n* = 21, 58.3%). The majority of reviews primarily focused on one specific type of disadvantage as the exposure. There were no reviews identified that included women involved in the judicial system, victims of modern slavery, homelessness or insecure housing, or experience of sex work.

A small number of reviews had considerable overlap in the primary studies (Supplementary Fig. 1, [19]), these were mostly reviews of interventions to prevent or treat mental health problems or group antenatal care interventions [22, 23, 36–38].

### Methodological quality

The AMSTAR-2 summary rating for the overall confidence of the results [18] was critically low in the majority of reviews(*n* = 21) (Table 3). Low quality ratings were most commonly due to lack of comprehensive literature search strategy (domain 4) or lack of reference to a registered, detailed protocol with justification for any deviation from this (domain 2).

**Table 3:** Methodological quality of the included systematic reviews assessed using AMSTAR-2

Author (year)	AMSTAR-2																Qualitative appraisal^a^
	**Item 1**	**Item 2**	**Item 3**	**Item 4**	**Item 5**	**Item 6**	**Item 7**	**Item 8**	**Item 9**	**Item 10**	**Item 11**	**Item 12**	**Item 13**	**Item 14**	**Item 15**	**Item 16**	
Asham (2017)	+	±	-	-	+	+	-	+	+	+	NM	NM	+	-	NM	+	Low
Boyle (2021)	+	-	-	-	-	-	-	-	-	-	NM	NM	-	-	NM	+	Critically low
Byerley (2017) [43]	-	-	-	±	+	-	-	-	+	-	NM	NM	+	-	NM	+	Low
Carter (2016) [28]	+	±	-	-	+	+	-	±	-	-	-	+	+	+	+	+	Critically low
Caulfield (2021)	+	+	+	+	+	+	+	+	+	-	NM	NM	+	+	NM	+	High
Coast (2016) [45]	+	-	-	-	-	-	-	±	-	-	NM	NM	+	+	NM	+	Critically low
Darling (2021) [44]	+	-	-	±	+	-	-	±	+	-	NM	NM	+	+	NM	-	Low
Eppes (2023) [51]	-	-	-	-	+	+	-	±	+	-	NM	NM	+	-	NM	+	Critically low
Essan (2022)	+	-	+	±	+	+	-	±	-	-	NM	NM	+	-	NM	-	Critically low
Fang (2022) [26]	+	-	-	±	+	+	-	±	+	-	+	-	+	+	+	+	Low
Friedrichson (2018)	+	-	-	-	-	-	-	±	-	-	NM	NM	-	-	NM	-	Critically low
Huang (2020) [27]	+	-	-	-	-	+	-	±	+	-	-	-	+	+	+	+	Critically low
Jahanfar (2014) [40]	+	±	-	+	+	+	+	+	+	-	NM	NM	+	+	NM	+	Moderate
Jidong (2021) [36]	-	±	-	±	+	-	-	±	+	-	NM	NM	-	-	NM	+	Low
Jongen (2014) [49]	-	-	+	+	-	+	-	±	-	-	NM	NM	-	-	NM	+	Critically low
Kaks (2020) [32]	-	-	-	-	+	-	-	-	+	-	NM	NM	-	+	NM	+	Critically low
Karger (2022) [46]	-	-	-	-	+	-	-	-	-	-	NM	NM	+	-	NM	+	Critically low
Kahn (2023)	+	±	-	+	+	+	-	-	-	-	NM	NM	-	-	NM	+	Critically low
Klatter (2022) [38]	-	-	-	-	+	-	-	±	+	-	NM	NM	-	+	NM	+	Critically low
Lieberman (2014) [35]	-	-	-	-	-	-	-	+	-	-	NM	NM	-	-	NM	-	Critically low
Lioret (2022)	+	+	+	±	+	-	-	-	+	-	NM	NM	-	-	NM	+	Low
Luo (2023) [42]	-	+	-	-	+	-	-	±	-	-	NM	NM	-	-	NM	+	Critically low
Martin Gomez (2022) [23]	+	±	+	+	+	+	-	+	+	-	+	+	+	+	+	+	Moderate
Mohammadi (2023) [30]	+	-	-	-	+	+	-	+	+	-	-	-	-	-	-	+	Critically low
Molloy (2020) [34]	+	+	+	-	+	+	+	±	+	+	NM	NM	+	-	NM	+	Low
Ponting (2020) [37]	+	+	-	±	+	+	-	+	+	-	NM	NM	-	-	NM	-	Low
Rivas (2015) [24]	+	+	-	+	+	+	+	+	+	+	+	+	+	+	NA*	+	High
Robinson (2018) [29]	+	-	-	-	+	+	+	±	-	-	-	-	-	+	-	-	Critically low
Rojas- Garcia (2014) [22]	+	-	-	+	+	-	-	±	-	-	+	+	+	+	+	-	Critically low
Sangsawang (2019) [41]	-	-	-	-	+	+	-	+	+	-	NM	NM	+	+	NM	+	Critically low
Seguera-Perez (2021)	-	±	-	-	+	+	-	-	+	-	NM	NM	+	-	NM	+	Low
Sukhato (2014)	+	-	-	-	+	+	-	±	+	-	+	-	+	+	+	+	Critically low
Terplan (2015) [25]	+	+	-	+	+	+	+	+	+	-	+	+	+	+	-	+	Moderate
Tibingana-Ahimbisibwe (2018)	-	-	-	-	+	+	-	±	-	-	NM	NM	+	+	NM	+	Critically low
Whittaker (2017)	+	+	+	+	+	+	+	+	+	-	+	+	+	+	+	+	High
Van Parys (2014) [39]	-	-	-	-	+	-	-	-	+	-	NM	NM	-	+	NM	-	Critically low

*NM* Not a meta-analysis therefore these criteria were not assessed

NA* insufficient number of studies for funnel plot

^a^One domain identified as critical by AMSTAR-2 guidance, on whether the review included justification for excluding individual studies (domain 7), was almost universally not performed and was excluded from the summary rating as the authors felt that it did not critically impair the quality of the review

### Quality of the evidence

We intended to report the quality of individual studies included in reviews, however 16 different tools were used to assess risk of bias. These tools used very different grading systems and therefore it was not possible to synthesise the quality of individual studies for this review (Supplementary Table S3). Very few reviews (*n* = 5) reported GRADE scores.

### Findings by Intervention type

The interventions were categorised as shown in Table 4, further detail on this is provided in Supplementary material. The majority of reviews used individual level interventions to improve maternal or infant / child health outcomes. In addition, interventions were categorised by the timing of intervention (pre-conception, pregnancy or postnatal) Supplementary Table S3.

**Table 4:** Level of change, operational definitions and examples of intervention categories

*Intervention Category*	*Operational Definition*	*Examples of interventions from selected primary studies included within reviews*
**Individual level of change:** influence behaviour through change in knowledge, attitudes, and beliefs, skills of women or influencing the people who closely interact with women, such as social support networks, or modifying the home / family environment		
**Home based interventions**	Education, psychosocial intervention, support or care delivered in the woman’s home by professional, peer or trained lay provider	Trained provider home visits through pregnancy and early childhood addressing prenatal and newborn care and maternal life skills [50] Nurse home visits from 25 weeks of pregnancy to 2 years of age on prenatal health, competent caregiving, and maternal self-sufficiency. [34]
**Psychosocial interventions**	Interpersonal or informational activities or strategies that target biological, behavioural, cognitive, emotional, or social factors, delivered to the individual or group in a clinic or community setting	Brief advocacy interventions delivered by bilingual domestic violence advocate offering support, referral. [24] Group cognitive behavioural or interpersonal therapy [37] Prenatal and postnatal group nutrition counselling delivered by a bilingual lactation consultant [53] Antenatal education by trained non-professional women in the community [48]
**Peer or lay support**	Lay or peer provider involved in range of activities such as advocacy, support, education	Peer led health advocacy program to ensure informed choice of healthcare for non-English speaking women [45] Interpersonal psychotherapy in group sessions led by peers [26] Individual phone-based peer support by mothers with a history of postpartum depression [26]
**Approaches to overcome physical barriers or incentivise health or healthcare**	Interventions that aim to overcome the physical barriers to health or health care such as vouchers, supplements, free baby equipment and transport	Provision of home equipment such as breast pump or supplemental nutrition support [53], Provision of childcare during weekly antenatal and relapse prevention groups [25], vouchers for drug free urine samples and bonus for perfect attendance [25]
**Written or digital education materials**	Information given in writing, or digital provision (e.g., website, app) as a standalone intervention	One-time social support intervention by pamphlet, video or video and pamphlet [41] 25-min video on breastfeeding [53]
(2) Organisational level of change- Change in physical environment, attitudes or knowledge of service providers, policies, regulations and culture of services		
**Integrated or interdisciplinary programs**	Interventions that support wider issues related to social determinants of health as well as pregnancy or postnatal care through integrating care or providing interdisciplinary services in one setting	Hospital based comprehensive interdisciplinary, adolescent specific prenatal and postnatal care program that provides psychiatric, social and nutritional services as an integrated program. [48] Teenage mother and child program with in-depth psychological and nutritional assessment, medical care, education about pregnancy, birth, contraception and infant care, and individual counselling on financial management, school and work. [54]
**Models of maternity care**	Organisational or service models for maternity care. This includes midwifery models where midwives are the central care provider providing continuity or caseload work and group care led by midwives or other healthcare professionals	Group antenatal care using centering pregnancy model [28] Midwifery models of care. [7]
**Interventions targeting cultural barriers to clinical care**	Strategies and interventions aiming to overcome cultural barriers to healthcare for example involving communities in designing and leading maternity services, education on cultural competency, navigator or link worker roles	Collaboration with indigenous communities to develop an integrated model of shared antenatal care including using aboriginal health workers and creating family friendly environments. [45] Lay link workers spanning hospital and community settings that work alongside health professionals as ‘facilitators’ and ‘interpreters’ while providing educational content. [45] Community controlled health service providing 1) transportation, informal childcare and home visits 2) female doctors and aboriginal health workers, cultural training for staff. [45] Community midwife and aboriginal health workers provide culturally appropriate services in the community with community development programs. [45]
(3) Community level of change—Influence community beyond the individual and immediate contacts through delivering community services, and spaces or creating shared identities and relationships between organisations / networks		
**Community engagement and development**	Interventions aiming to influence community beyond the individual and immediate contacts, through engaging community in designing and/or leading services or community development activities	Community controlled health service providing 1) transportation, informal childcare and home visits 2) female doctors and aboriginal health workers, cultural training for staff. [45] Community wide health education and events and environment modification including changing to low sugar drinks at events. [50]
**Media Campaign**	Interventions aiming to influence community through education	Breastfeeding media campaign aiming to promote breastfeeding to indigenous communities. [50] Community public service announcements, video, billboard and infant shirts within a broader intervention of health system design and individual education and support. [50]
(4) Environment, Policy level of change—Influence policy, advocacy, wider environments and structures that impact on health		
**Policies**	A system or institution wide regulation or process intervention	Baby friendly hospital initiatives. [53] Expansion of universal healthcare policies. [52] Policies to limit pacifier use and promote skin to skin in hospital setting. [53]

## Individual

### Home based interventions

Four narrative reviews included primarily home-visiting interventions. In women with socioeconomic disadvantage, nurse or social worker led sustained home-visiting programs had some benefit to breastfeeding practices [33, 34], anxiety, immunisation compliance, and preventing child injuries or maltreatment, but had no statistically significant effect on: LBW [33, 34], PTB, postnatal health, common childhood illnesses, family planning, infant mortality, [34] or infant anthropometric measurements. [33] Peer led or trained provider home-visiting programs were of some benefit to increasing breastfeeding initiation, attendance at antenatal education or care, reducing LBW, PTB and complications during delivery. Minimal benefit in improving maternal mental health was described. [32] Nurse led home visits, targeted to prevent and reduce intimate partner violence (IPV) in the postnatal period, showed mixed [31] or neutral effects [40] from a small number of studies.

There were six further reviews combining home-visiting with other interventions, such as small group school education sessions or screening and referrals. In indigenous women, breastfeeding outcomes were again improved, with some benefit in reducing LBW, and child weight, but no convincing evidence of a beneficial effect on maternal nutrition or weight [50]. However, a review of predominantly peer or trained lay breastfeeding interventions in women of ethnic minority in the USA, found few studies impacted breastfeeding beyond the early postpartum [53]. In adolescent mothers, home-visiting interventions had limited effectiveness in preventing postpartum depression [35, 41], PTB or LBW [20, 48] from a small number of primary studies. Home-visits, mainly delivered by peers or community mentors with a focus on community services, practical support, education and employment had significant benefit in preventing repeat births (RR0.6, 0.39 to 0.9, GRADE strength of evidence: moderate) and repeat unintended pregnancy (RR of 0.88 (95% CI 0.78 to 1.00)), but not increasing use of contraception(Aslam et al., 2017) [21]. A summary of the evidence base for each category of intervention is shown in Table 5.

**Table 5:** Summary of potential benefit of interventions

Intervention Type	Benefit or potential benefit	Limited or no benefit
Home-based interventions [20, 21, 31–35, 40, 41, 48, 50, 53]	Potential benefit on some maternal and child health outcomes in women with disadvantage Good evidence for preventing repeat births and repeat unintended pregnancy in adolescents	Limited benefit for preventing or reducing IPV
Psychosocial interventions [20, 22–25, 35–37, 40–42, 48, 53]	Potential benefit in treating perinatal depression in adolescents and treating and preventing perinatal depression in disadvantaged women from mainly minoritized ethnic groups Potential to improve breastfeeding in women from various minoritized ethnic groups Advocacy interventions are of potential benefit in preventing and reducing IPV Potential to benefit neonatal outcomes in adolescents	Limited role in treating depression in migrant women Non-advocacy-based psychosocial interventions not effective at reducing IPV In women with substance abuse, limited benefit in improving neonatal health outcomes
Peer support interventions [26, 27, 33, 48]	Insufficient evidence	
Approaches to overcome physical barriers or incentivise health or healthcare. [21, 25, 45, 46, 49, 53]	Insufficient evidence	
Digital and written education interventions [33, 35, 36, 41, 51, 53]	-	Limited benefit at improving maternal health outcomes in vulnerable families
Models of maternity care [7, 28–30, 43, 44]	In women with social disadvantage, midwife models of care had mostly positive effects across a range of outcomes Group care identified to have potential to benefit PTB and breastfeeding rates in African American women	Mixed impact of group care on adolescents and women with low-income
Integrated or Interdisciplinary care [7, 38, 42, 48, 50, 54]	In women with social disadvantage, interdisciplinary care had potential benefit across a range of outcomes	Limited evidence of interdisciplinary programs at reducing repeat teenage pregnancy or improving neonatal outcomes
Interventions targeting cultural barriers to clinical care [13, 21, 22, 24, 30, 39–42, 45, 46, 49, 50, 53]	Some evidence that cultural interventions improved use, or timing of care but low quality	Mixed impact across other outcomes
Community engagement and development [45, 46, 49, 50]	Insufficient evidence	
Media Campaigns [50]	Insufficient evidence	
Policies [52, 53]	Insufficient evidence	

### Individual Psychosocial interventions

Seven reviews reported efficacy of psychosocial interventions in preventing or treating depression. In adolescent mothers, psychosocial interventions (phone-based motivational interviewing or interpersonal group intervention) were effective at treating perinatal depression, although effects were not sustained and the studies were methodologically weak. Efficacy in preventing depression in adolescent mothers was positive [41] or mixed [35], with no consistent characteristics across efficacious interventions.

Similarly in women with social disadvantage that were primarily of minoritised ethnicity, two partially overlapping reviews concluded that psychosocial interventions were effective in treating [22] and preventing [22, 23] depression, with a smaller significant impact reported from the higher quality meta-analysis ( SMD-0.197, 95% CI -0.339, -0.054) [23]. A range of individual and group IPT or CBT were delivered during pregnancy, or the postnatal period. The characteristics of efficacious interventions varied [22, 23]. In Black African or Caribbean women, individual psychosocial interventions improved depression symptoms or mood regulation. Interventions that enhanced parenting confidence and self-care were reported as most effective, with cultural adaptation being key [36]. In comparison, predominantly group interventions in Latina and Black women had limited evidence of effectively reducing depressive symptoms. CBT was the modality with most evidence. Only two studies evaluated anxiety symptoms, with no benefit [37]. In migrant women, limited studies, of predominantly group psychosocial interventions, delivered by culturally appropriate trained providers, had limited evidence of effectiveness in improving maternal depression [42].

In varied minoritised ethnic groups in the USA, the majority of psychosocial interventions showed benefit to at least one breastfeeding outcome. Interventions were varied, including group cooking classes, in person or telephone breastfeeding education and support, and motivational interviewing by peers or trained professionals [53].

In women with, or at risk of, IPV, two partially overlapping Cochrane reviews reported some benefit of advocacy interventions, but heterogenous study designs and interventions precluded meta-analysis [24, 40]. In women with, or at risk of, IPV, other psychosocial interventions (individual or group CBT or IPT), were not effective at reducing IPV [40].

Few studies of women receiving treatment for substance misuse reported maternal or neonatal health outcomes. Addition of positive reinforcement (e.g., financial incentives for drug free screens or peer reinforcement) or motivational techniques to usual care (ranging from brief intervention to group or individual counselling with coordinated obstetric care and on-site childcare) did not significantly reduce PTB (RR 0.71,95% CI 0.34 to 1.51, GRADE: moderate), LBW (RR 0.72, 95% CI 0.36 to 1.43, GRADE: moderate) or adverse perinatal events (*p* = 0.22) [25]. However, neonates remained in hospital for fewer days in positive reinforcement intervention groups (RR -1.27, 95% CI -2.52 to -0.03, GRADE: moderate) [25].

In adolescents, a broad range of tailored interventions, including nutritional assessment with dietary counselling, a voluntary teenage parenting program aiming to provide support systems, and antenatal education in the community were reported to mostly benefit rates of PTB and LBW [48]. However, a further review found limited efficacy of education programs for these outcomes [20].

### Peer support interventions

From two reviews which included five individual primary studies meeting our criteria [26, 27] peer support had inconsistent effects in treating depression [26, 27]. A small number of primary studies from other reviews suggested some benefit on attendance at antenatal care, PTB and LBW in adolescent pregnancies [48], and breastfeeding practice in indigenous women [33]. However, mostly no impact on care engagement in minoritized ethnic groups [45].

### Approaches to overcome physical barriers or incentivise health or healthcare

The majority of interventions which included approaches to overcome physical barriers to healthcare combined this with multi-component interventions, for example by provision of transport within antenatal care programs for indigenous women [45, 46, 49] or vouchers and transport within a program of support for mothers with substance misuse [25]. A small number of single studies utilised other approaches, such as vouchers to incentivise breastfeeding with breastfeeding support services [53] and incentives to attend week peer group meetings with free contraceptives and information on jobs for adolescent mothers [21]. Other single studies provided breast pumps [53], a pregnancy basket with leaflets, baby toiletries and a safe sleeper with fresh fruit and food vouchers [50].

### Digital and written education interventions

From a small number of studies, there was minimal evidence of efficacy of digital or written education on weight and feeding outcomes. One review with six relevant studies of digital media platforms to improve health behaviour in vulnerable families, reported minimal or no benefit of: one-way texts on gestational weight gain or infant weight, two-way text exchange on breastfeeding and depression and only benefit of an interactive app on weight loss for low income, overweight, mothers if there was high engagement [51]. In ethnic minority women in the USA, a digital app to identify breastfeeding champions, two-way text service, and written or video information, did not show significant impact on breastfeeding outcomes [53]. Other reviews included single studies with varied digital interventions including social media groups to improve mental health [36], or prevent childhood obesity [33], or social support interventions by a combination of pamphlet or video [35, 41] or educational videos to promote breastfeeding [33].

## Organisational

### Models of maternity care

In women with social disadvantage, midwife models of care had mostly positive effects across a range of outcomes. The majority of studies reported benefit to breastfeeding rates and antenatal care coverage, with some benefit in reducing PTB and LBW. Effects on perinatal mortality and uptake of childhood immunisations were less positive [7]. Some of these studies of midwifery models of care were tailored to the specific needs of the community or delivered in a community setting, with similar effectiveness [7].

Two reviews reported specifically the efficacy of group care (midwife led alone or with other providers) [28, 43]. For adolescents, findings across nine primary studies were inconsistent, with benefit to antenatal attendance, uptake of long-acting contraception, depressive symptoms and breastfeeding, but a mixed effect on LBW, PTB, and rapid repeat pregnancy, and no benefit to NICU admission. For women with low-income, results were also inconsistent. The majority of studies demonstrated benefit to PTB, but not consistently to other neonatal outcomes, including LBW. Mostly single studies reported benefit to initiation of breastfeeding, attendance at care and rates of rapid repeat pregnancy. A single, non-randomised study, reported that in women with opioid addiction, group care had no significant difference in neonatal outcomes but did improve attendance [43].

In African American women, group care reduced rates of PTB, which was significant when limited to high quality studies (*n* = 8% vs. 11.1%, pooled RR 0.55, 95% CI 0.34–0.88). The effect was not observed in Latina women (5.9% vs. 4.7%%, pooled RR 1.66, 95% CI 0.66–4.18), [28]. Several other studies included a high proportion of women from minoritised ethnic groups but had more mixed impacts on PTB [43], but increased breastfeeding [43]. Similarly a third meta-analysis in minoritised ethnic groups, including many of the same studies, concluded that group care improved breastfeeding initiation by 53% (95% CI 29%-81%) and among African American participants by 71% (95% CI 27%-131%) [29].

Two additional reviews, which predominantly overlapped and combined models of care with varied clinical antenatal interventions (e.g., nurse telephone intervention, multidisciplinary care or nurse home visit) presented similar or more reserved benefit to PTB and other neonatal outcomes in women with social disadvantage [30, 44].

### Integrated or interdisciplinary care

In women with social disadvantage, interdisciplinary care was reported to mostly benefit infant mortality, LBW, breastfeeding and attendance at antenatal care with some benefit to PTB and perinatal mortality but not uptake of contraception. There were few studies which targeted interdisciplinary care to the needs of communities but from these, similar mixed effects were reported [7].

Three reviews evaluated interventions in adolescents that integrated education, counselling and housing or financial management with varied medical services, case management and referrals [38, 48, 54]. There was no significant benefit to rates of repeat teenage pregnancy following community (RR 1.00 (95% CI 0.65 to 1.52 (GRADE: moderate) or telephone programs (RR 0.89 (95% CI 0.55 to 1.46) (GRADE: moderate). Similarly, in a lower rated review one of four primary studies had significant benefit in reducing repeat teen pregnancy. Single primary studies reported that maternal morbidity, contraception uptake, postnatal attendance and baby immunisation rates all improved [54]. In comparison, comprehensive interdisciplinary interventions, delivered in hospitals, schools, or communities improved attendance at antenatal care in most studies, but the effect on PTB and LBW were inconsistent. [48] The extent to which psychosocial interventions were collaborative with multiprofessional involvement did not improve efficacy in managing depression symptoms [38]. Single primary studies evaluated integrated care pathways for indigenous women [50] and women that migrated prior to birth [42].

### Interventions targeting cultural barriers to clinical care

One review of cultural interventions identified 13 relevant studies delivered by culturally appropriate professionals or peers [45]. Most were methodologically weak but demonstrated significant improvement in at least one outcome around use, or timing of, antenatal care or postnatal care. Half of moderate quality studies found no significant benefit. In Australia, effective interventions included indigenous staff, community control and/or participation and either outreach or a community setting which was culturally friendly. In the USA effective interventions included lay or trained individuals who shared a language or cultural characteristics with the target population and provided a range of educational and support outreach [45].

However, in indigenous communities, multi-component interventions which included maternity care with the addition of health education / brief interventions, coordination of care, and community leader support [49], and culturally adapted maternity care [46] had inconsistent effects on neonatal outcomes [46, 49], and non-significant benefit to antenatal care attendance, breastfeeding and childhood immunisation uptake [49]. A review of five varied interventions which all included cultural adaptation of integrated or community based antenatal care programs identified mostly benefit to BF initiation and duration and PTB but not LBW. For example, native American talking circles within a multi-component breastfeeding intervention. [50] Several other reviews included primary studies of interventions with a strong focus on meeting cultural needs. Results showed limited effectiveness in preventing depression in adolescent mothers [41], improving mental health in migrant women [42], treating depression and anxiety in Latina and Black women [37] and treating depression in socially disadvantaged women [22]. A small number of primary studies used culturally adapted interventions to prevent PTB in disadvantaged women [30], reduce IPV [24, 39, 40], improve infant outcomes, iron deficiency and breastfeeding [53] in women of ethnic minority [13] and prevent repeat unintended teenage pregnancy [21].

## Community

### Community engagement and development

Whilst no reviews focused specifically on community interventions, many included primary studies where this was a key part of the intervention. For example, in a review of culturally appropriate maternity care [45], six studies had a substantial community focus. The majority improved antenatal attendance. Community control or setting were identified as key strategies of success. In indigenous women, a further review identified from single studies, significant benefit to childhood weight and some benefit to breastfeeding, but no benefit to maternal weight or dental decay [50]. Further single studies included comprehensive shared care delivered in a mobile bush clinic with sexual health, domestic violence counselling and health education [49] and a community driven home visiting program [46].

### Media campaigns

Media campaigns, all in conjunction with broader interventions, did not statistically benefit a broad range of maternal and child nutrition outcomes [50].

### Environment and policy interventions

Few studies evaluated environmental or policy level interventions. The special supplemental nutrition program for women, infants and children (WIC) delivers supplemental foods, education, breastfeeding support and referrals to low-income, nutritionally at risk pregnant and postpartum women and children. From a small number of primary studies WIC participation lowered risk of PTB, LBW, and infant mortality. Evidence was less strong but also suggested increased child immunisations, but not breastfeeding initiation [52].

Federal food assistance policies may have a small positive effect on breastfeeding outcomes in minoritised ethnic groups in the USA. Baby Friendly Initiatives and different maternity care policies reported positive impact on breastfeeding practices until discharge from hospital, but with mixed effects in the early postnatal period. Interventions not specifically targeted to breastfeeding (e.g., commercial insurance) showed no benefit [53].

### Lived experience team impression of the evidence

**Fig. 2:**
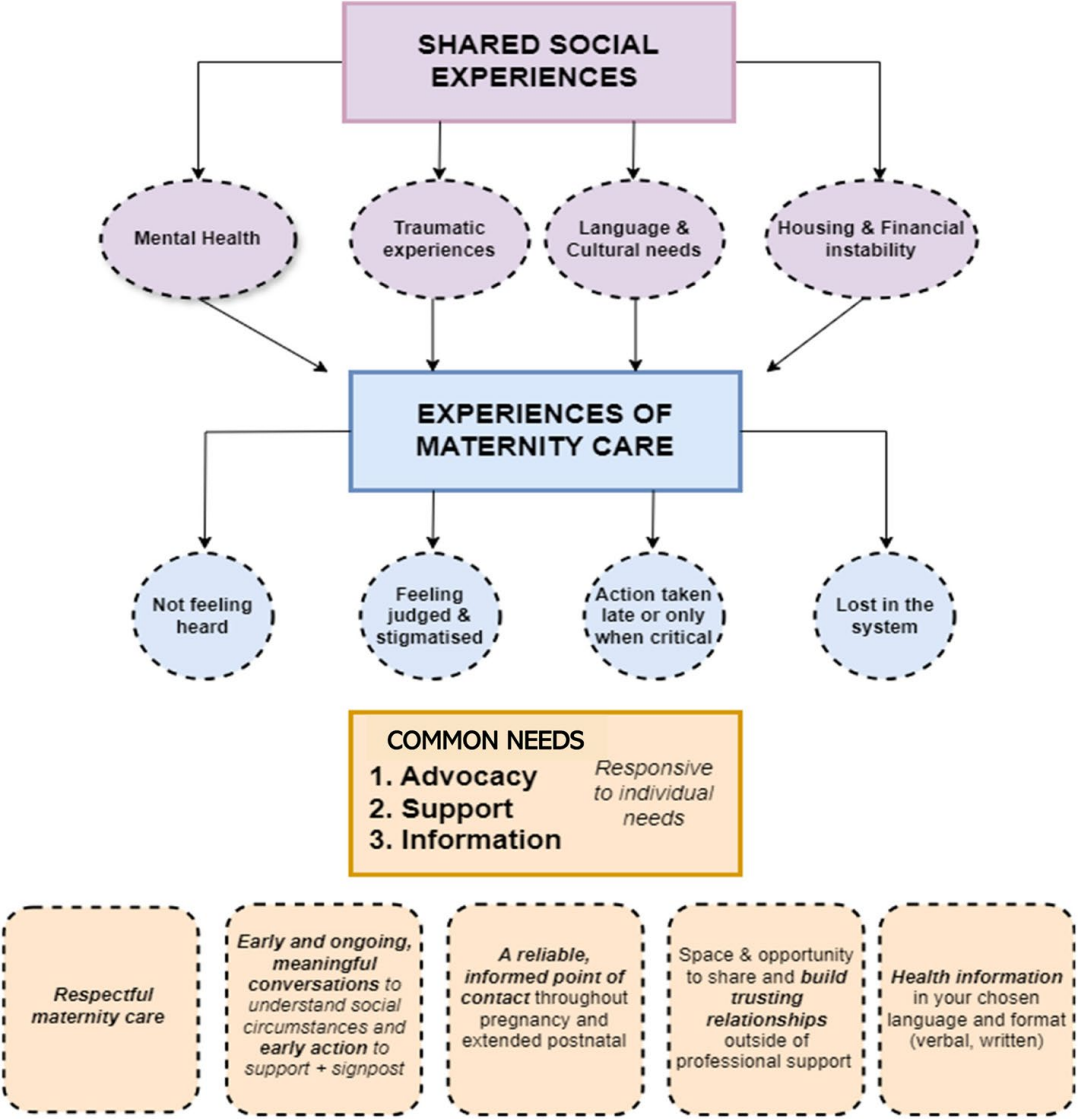
Reflections from women with lived experience of multiple social disadvantages in pregnancy on the evidence base in relation to their experiences

The process of involving lived experience in this review is described in Panel 1. The experiences of our lived experience group, the relevance of identified interventions to their experiences and identified gaps in the evidence are shown in Fig. 2.

**Table:** 

**Panel 1: Involving women with lived experience in interpreting the evidence**
We held two group discussions, of two hours each, with six women with recent experience of pregnancy. The women had experiences of multiple disadvantage and inequalities, including insecure housing, financial deprivation, substance misuse, recent migration, minoritized ethnicity and domestic abuse. The group were identified through Birth Companions, a charity that supports women with inequalities and disadvantage in the first 1001 days. Birth Companions supported the women to participate through being a trusted figure to encourage open sharing, offering a break out space during sessions and follow up support if required. The group were paid in lines with current guidance from the National Institute for Health and Care Research. This included costs for childcare. The sessions were held online, at the request of the group.
In the first session the group explored how their social circumstances impacted on their experiences of care during pregnancy and the year after birth. This was held after the search was undertaken but prior to synthesis of the results. In the second session the group explored the ways in which their experiences around pregnancy could have been improved, the extent to which the interventions identified in this review were relevant to their experiences, and whether other interventions types would have been preferred. This was held after the main intervention types had been synthesised.
The group identified common themes that would have improved their experiences: respectful maternity care, early and ongoing conversations to identify social needs and action to support them, a reliable informed point of contact, space and opportunity to build trusted relationships and accessible health information. They felt these could have been achieved through a variety of intervention types identified in the review, including mental health support, models of midwifery care and peer support. However, women had different preferences as to how these needs could be met depending on their experiences. This emphasises the need for personalised care. There were key experiences such as the impact of traumatic experiences and feeling judged and stigmatised, that were not reflected in the existing literature of interventions. The impact of insecure housing and financial instability on experience of pregnancy were significant and rarely felt to be adequately addressed in the identified interventions. The group contribute to the development of Figure 3.

## Discussion

We included 36 systematic reviews and meta-analyses of interventions aiming to improve maternal and child health outcomes in women with social disadvantage in pregnancy. The majority were low or critically low quality, undertaken in North America and included individual level interventions. Overall, there was potential benefit of home visiting programs. Individual psychosocial interventions were predominantly reported to be effective at treating depression. Midwife models of care had mostly positive effects across a range of outcomes. Evidence for group models of antenatal care were more mixed. The majority of reviews of integrated or interdisciplinary care were in adolescent mothers, with mixed effects. There was limited evidence of interventions aiming to overcome physical barriers or incentivise healthcare, these were largely combined with comprehensive multicomponent interventions. Cultural adaptation and involvement of communities in design and delivery of interventions were frequently described as key, but there was limited or inconsistent evidence of efficacy, especially in Europe. We identified few reviews of policy interventions. No reviews included preconception interventions, or interventions for women involved in the judicial system, that are victims of modern slavery, experiencing homelessness or insecure housing or experience of sex work and few included women with substance misuse.

The main strengths of this overview of reviews are demonstrating the breadth of interventions, their efficacy across varied exposures and outcomes and describing the quality of research in this field. The robust search strategy and extensive grey literature review is a strength. However, the nature of including only systematic reviews means that some promising interventions that have not been examined in systematic reviews will not be included. For example, we identified few reviews of policies. This may be because they are evaluated in study types or published on platforms not typically included in reviews. One example, Sure Start, an intervention which provided a comprehensive early years program, was shown to have a significant impact on childhood academic performance in England using policy evaluation methodology [55]. A recent policy mapping review has identified relevant UK early years policies, and could be used in conjunction with our umbrella review [56].

Where socioeconomic deprivation was the sole criteria for eligibility of the population in the review, we included studies where deprivation was assessed for individual women and not based solely on regional or area measures. This was on the basis that this would be more likely to be known to clinicians and may inform changes in practice, however some relevant literature may have been excluded. This study is also limited to exploring the quantitative effect of interventions on health outcomes. This simple perspective was selected to make the umbrella review feasible and provide a clear resource for health care providers and policymakers. However, inclusion of qualitative research is vital to understand which components of multifaceted interventions are effective, and how they work and interact in different settings [57, 58].

Women experience multiple disadvantages that interact and accumulate, therefore since the purpose of this review was to allow this complexity, the heterogeny in exposure and interventions identified was expected. However, this means there is overlap in intervention types and population groups. In addition, the size of the review meant there was inadequate capacity to go back to the primary studies and findings are reliant on information provided in the systematic reviews and meta-analyses.

Involving women with lived experience of social disadvantage was essential to accelerate understanding of experiences and relevance of possible interventions. Common experiences were identified and several of the identified interventions were reported to be relevant to improving these experiences. The impact of traumatic experiences and feeling judged and stigmatised were other key themes identified by women with lived experience that were not reflected in the existing literature of interventions. In keeping with umbrella reviews of interventions to improve health of non-pregnant socially excluded people [59], structural factors such as housing and employment were important to women but there was an absence of interventions identified. Future research is required to explore interventions that may meet these gaps, as well as for women with multiple interacting social disadvantages.

Inequalities in maternal mortality and morbidity exist in the UK [1], USA and Europe [60]. Whilst a broad range of interventions were identified, and some showed potential benefit, overall, there is limited, high-quality evidence undertaken in Europe. Therefore, despite it being a current policy priority, current evidence is insufficient to inform practice in Europe, without additional work to understand the relevance and cost effectiveness of potentially effective interventions.

## Conclusions

This umbrella review identified potential benefit of home-based interventions, psychosocial interventions, models of midwifery care and interdisciplinary programs of care for some population groups, across a range of maternal and child health outcomes. However, the majority of reviews were of critically low or low quality, did not encompass the complexity of social disadvantage and some at risk populations were excluded from all reviews. The majority of interventions were primarily aimed at influencing individual women’s behaviour through changing knowledge, attitudes and beliefs, and there were few interventions aimed at the environmental or policy level. This means there is an absence of high-quality evidence, especially of interventions that impact of underlying determinants of health such as housing and financial resource, and on access to care, quality, and experience of care. Interventions which target only women with the lowest incomes will not improve outcomes for the greater number of women with social disadvantage that fall above the threshold for intervention. Therefore, while local and national governmental action is required improve the upstream determinants of health, maternity as a universal service, has a role in adapting the scale and intensity of its offer to mitigate the impact of these determinants on maternal and child health outcomes. Future high-quality research is required to explore ways of doing this, for example through culture- and trauma-informed care, and alternative models or place of care which integrate wider community services.

## Supplementary Information

Supplementary Material 1.

## Data Availability

No datasets were generated or analysed during the current study.

## Abbreviations

PTB: Preterm birth
LBW: Low Birth Weight
