# Supplementary material for: Interventions to reduce inequalities for pregnant women living with disadvantage in high-income countries: an umbrella review
Source: BMC Public Health. 2025 Mar 25;25:1140. doi: 10.1186/s12889-025-22283-5 (PMC11938774; doi:10.1186/s12889-025-22283-5)
Supplement: Supplementary file 1 — Supplementary Material 1. [file 12889_2025_22283_MOESM1_ESM.docx]

**Table S1.** PRIOR checklist (Preferred Reporting Items for Overviews of Systematic Reviews) (Gates et al., 2022; Bougioukas et al., 2019)

| Section topic | Item No | Item | Location where item is reported |
| --- | --- | --- | --- |
| **Title** | | |  |
| Title | 1 | Identify the report as an overview of reviews. | Page 1 Line 2 |
| **Abstract** | | |  |
| Abstract | 2 | Provide a comprehensive and accurate summary of the purpose, methods, and results of the overview of reviews. | Page 2 |
| **Introduction** | | |  |
| Rationale | 3 | Describe the rationale for conducting the overview of reviews in the context of existing knowledge. | Page 3-4 |
| Objectives | 4 | Provide an explicit statement of the objective(s) or question(s) addressed by the overview of reviews. | Page 4 line 103-105 |
| **Methods** | | |  |
| Eligibility criteria | 5a | Specify the inclusion and exclusion criteria for the overview of reviews. If supplemental primary studies were included, this should be stated, with a rationale. | Table 1 |
|  | 5b | Specify the definition of “systematic review” as used in the inclusion criteria for the overview of reviews. | Page 5 Line 120 |
| Information sources | 6 | Specify all databases, registers, websites, organisations, reference lists, and other sources searched or consulted to identify systematic reviews and supplemental primary studies (if included). Specify the date when each source was last searched or consulted. | Page 5 Line 126-129 and Table S2 |
| Search strategy | 7 | Present the full search strategies for all databases, registers and websites, such that they could be reproduced. Describe any search filters and limits applied. | Table S2 |
| Selection process | 8a | Describe the methods used to decide whether a systematic review or supplemental primary study (if included) met the inclusion criteria of the overview of reviews. | Page 5 line 132-133 |
|  | 8b | Describe how overlap in the populations, interventions, comparators, and/or outcomes of systematic reviews was identified and managed during study selection. | Page 6 line 143-144 |
| Data collection process | 9a | Describe the methods used to collect data from reports. | Page 5 line 132-134 |
|  | 9b | If applicable, describe the methods used to identify and manage primary study overlap at the level of the comparison and outcome during data collection. For each outcome, specify the method used to illustrate and/or quantify the degree of primary study overlap across systematic reviews. | Page 6 line 144-146 |
|  | 9c | If applicable, specify the methods used to manage discrepant data across systematic reviews during data collection. | Page 6 line 148-149 |
| Data items | 10 | List and define all variables and outcomes for which data were sought. Describe any assumptions made and/or measures taken to identify and clarify missing or unclear information. | Table 2 |
| Risk of bias assessment | 11a | Describe the methods used to assess risk of bias or methodological quality of the included systematic reviews. | Page 5 line 136-138 |
|  | 11b | Describe the methods used to collect data on (from the systematic reviews) and/or assess the risk of bias of the primary studies included in the systematic reviews. Provide a justification for instances where flawed, incomplete, or missing assessments are identified but not reassessed. | Page 10 line 209-212 |
|  | 11c | Describe the methods used to assess the risk of bias of supplemental primary studies (if included). | NA |
| Synthesis methods | 12a | Describe the methods used to summarise or synthesize results and provide a rationale for the choice(s). | Page 6 line 143-144 |
|  | 12b | Describe any methods used to explore possible causes of heterogeneity among results. | NA – narrative |
|  | 12c | Describe any sensitivity analyses conducted to assess the robustness of the synthesised results. | NA – narrative |
| Reporting bias assessment | 13 | Describe the methods used to collect data on (from the systematic reviews) and/or assess the risk of bias due to missing results in a summary or synthesis (arising from reporting biases at the levels of the systematic reviews, primary studies, and supplemental primary studies, if included). | Protocol publication and Page 10 209-212 |
| Certainty assessment | 14 | Describe the methods used to collect data on (from the systematic reviews) and/or assess certainty (or confidence) in the body of evidence for an outcome. | Protocol publication and age 10 line 212 |
| **Results** | | |  |
| Systematic review and supplemental primary study selection | 15a | Describe the results of the search and selection process, including the number of records screened, assessed for eligibility, and included in the overview of reviews, ideally with a flow diagram. | Figure 1 |
|  | 15b | Provide a list of studies that might appear to meet the inclusion criteria, but were excluded, with the main reason for exclusion. | Figure 1 |
| Characteristics of systematic reviews and supplemental primary studies | 16 | Cite each included systematic review and supplemental primary study (if included) and present its characteristics. |  |
| Primary study overlap | 17 | Describe the extent of primary study overlap across the included systematic reviews. | Figure 2 |
| Risk of bias in systematic reviews, primary studies, and supplemental primary studies | 18a | Present assessments of risk of bias or methodological quality for each included systematic review. | Table 3 |
|  | 18b | Present assessments (collected from systematic reviews or assessed anew) of the risk of bias of the primary studies included in the systematic reviews. | Page 10 line 209-212 |
|  | 18c | Present assessments of the risk of bias of supplemental primary studies (if included). | NA |
| Summary or synthesis of results | 19a | For all outcomes, summarise the evidence from the systematic reviews and supplemental primary studies (if included). If meta-analyses were done, present for each the summary estimate and its precision and measures of statistical heterogeneity. If comparing groups, describe the direction of the effect. | Page 18-20 |
|  | 19b | If meta-analyses were done, present results of all investigations of possible causes of heterogeneity. | NA |
|  | 19c | If meta-analyses were done, present results of all sensitivity analyses conducted to assess the robustness of synthesised results. | NA |
| Reporting biases | 20 | Present assessments (collected from systematic reviews and/or assessed anew) of the risk of bias due to missing primary studies, analyses, or results in a summary or synthesis (arising from reporting biases at the levels of the systematic reviews, primary studies, and supplemental primary studies, if included) for each summary or synthesis assessed. | Page 10 line 209-212 |
| Certainty of evidence | 21 | Present assessments (collected or assessed anew) of certainty (or confidence) in the body of evidence for each outcome. | GRADE results presented where available through pages 18-20 |
| **Discussion** | | |  |
| Discussion | 22a | Summarise the main findings, including any discrepancies in findings across the included systematic reviews and supplemental primary studies (if included). | Page 22, paragraph 1 |
|  | 22b | Provide a general interpretation of the results in the context of other evidence. | Page 22 paragraph 2&3 |
|  | 22c | Discuss any limitations of the evidence from systematic reviews, their primary studies, and supplemental primary studies (if included) included in the overview of reviews. Discuss any limitations of the overview of reviews methods used. | Page 22 line 495-499 and page 23 line 500-508 |
|  | 22d | Discuss implications for practice, policy, and future research (both systematic reviews and primary research). Consider the relevance of the findings to the end users of the overview of reviews, e.g., healthcare providers, policymakers, patients, among others. | Page 23 521-527 and page 23 527 to 24 540 |
| **Other information** | | |  |
| Registration and protocol | 23a | Provide registration information for the overview of reviews, including register name and registration number, or state that the overview of reviews was not registered. | Page 2 line 42 and page 4 line 109 |
|  | 23b | Indicate where the overview of reviews protocol can be accessed, or state that a protocol was not prepared. | Page 4 line 111 |
|  | 23c | Describe and explain any amendments to information provided at registration or in the protocol. Indicate the stage of the overview of reviews at which amendments were made. | Table 1, page 10 line 209 |
| Support | 24 | Describe sources of financial or non-financial support for the overview of reviews, and the role of the funders or sponsors in the overview of reviews. | Page 1 line 23-24 |
| Competing interests | 25 | Declare any competing interests of the overview of reviews' authors. | Page 1 line 20-21 |
| Author information | 26a | Provide contact information for the corresponding author. | Page 1 |
|  | 26b | Describe the contributions of individual authors and identify the guarantor of the overview of reviews. | Page 24 line 550 |
| Availability of data and other materials | 27 | Report which of the following are available, where they can be found, and under which conditions they may be accessed: template data collection forms; data collected from included systematic reviews and supplemental primary studies; analytic code; any other materials used in the overview of reviews. | Page 24 line 545-546 |

**Supplementary Table 2: Search strategy**

[Medline (Ovid MEDLINE® Epub Ahead of Print, In-Process & Other Non-Indexed Citations, Ovid MEDLINE® Daily and Ovid MEDLINE®) 1946 to present](https://ovidsp.ovid.com/ovidweb.cgi?T=JS&NEWS=N&PAGE=main&SHAREDSEARCHID=42KZG6wV5VYVXpeZUgOn1iMiAJ39KJD4jN1dEvu9sB3mj26Z5mRNxMbpWRpRwrZRm)

| \|  \|  \| \| --- \| --- \| \| 1 \| intersectional framework/ \| \| 2 \| Socioeconomic Factors/ \| \| 3 \| Vulnerable Populations/ or Minority Groups/ \| \| 4 \| social vulnerability/ \| \| 5 \| sociodemographic factors/ \| \| 6 \| Healthcare Disparities/ \| \| 7 \| economic factors/ or economic stability/ or housing instability/ or exp ill-housed persons/ or economic status/ or poverty/ or poverty areas/ or social class/ or low socioeconomic status/ or social mobility/ or social factors/ or working poor/ \| \| 8 \| social isolation/ \| \| 9 \| domestic violence/ or intimate partner violence/ or spouse abuse/ or battered women/ \| \| 10 \| exp educational status/ or employment/ or unemployment/ \| \| 11 \| sex workers/ or sex work/ \| \| 12 \| human traffiking/ or enslaved persons/ \| \| 13 \| crime/ or criminals/ or prisoners/ or criminal law/ or prisons/ \| \| 14 \| deportation/ or refugees/ or exp "emigrants and immigrants"/ \| \| 15 \| drug users/ \| \| 16 \| exp indigenous peoples/ or roma/ or medically uninsured/ or exp asian people/ or exp black people/ or caribbean people/ \| \| 17 \| (socioeconomic? or socio-economic? or sociodemographic? or socio-demographic? or psychosocial or psycho-social).ti,kf. \| \| 18 \| ((socioeconomic? or socio-economic? or sociodemographic? or socio-demographic? or psychosocial or psycho-social or social or economic) adj3 (factor? or risk? or status or level?)).ab. \| \| 19 \| ((social or economic) adj3 (disparit* or disadvantag* or vulnerab* or depriv* or hardship)).ti,ab,kf. \| \| 20 \| (social* adj2 isolat*).ti,ab,kf. \| \| 21 \| social determinants of health.ti,ab,kf. \| \| 22 \| ((disadvantag* or vulnerab* or depriv* or hardship) adj3 (wom?n or population? or people or communit* or neighbo?rhood? or area? or family or families)).ti,ab,kf. \| \| 23 \| ((poor or poverty) adj3 (wom?n or population? or people or communit* or neighbo?rhood? or area? or family or families)).ti,ab,kf. \| \| 24 \| (homeless* or couchsurf* or couch surf* or (hous* adj3 (instability or unstab* or uncertain*))).ti,ab,kf. \| \| 25 \| (income adj3 (low* or level? or status)).ti,ab,kf. \| \| 26 \| (((education* or academic) adj3 (level? or status or achievement or attainment)) or (numeracy or literacy)).ti,ab,kf. \| \| 27 \| ((spous* or domestic or intimate partner) adj2 (abuse* or violence)).ti,ab,kf. \| \| 28 \| (sex work* or prostitut*).ti,ab,kf. \| \| 29 \| (modern slave* or (enslav* adj2 (worker? or people or person? or population? or wom?n))).ti,ab,kf. \| \| 30 \| (criminal* or prison* or incarcerat* or imprison* or probation* or detention centre? or detention center? or jail? or gaol?).ti,ab,kf. \| \| 31 \| (refugee? or asylum seeker? or migrant? or immigrant? or deportation).ti,ab,kf. \| \| 32 \| ((black? or afrocarib* or afro-carib* or african american or latino? or latina? or latinix or hispanic or asian or indigenous or aborigin* or roma or gypsy or traveller) adj2 (people or person or population or wom?n or family or families or communit*)).ti,ab,kf. \| \| 33 \| (ethnic* or minorit* or race or racial).ti,kf. \| \| 34 \| ((ethnic* or minorit* or racial) adj2 (group? or people or person or population or wom?n)).ab. \| \| 35 \| (complex* adj3 (need? or risk? or factor?)).ti,ab,kf. \| \| 36 \| or/2-35 \| \| 37 \| Pregnancy/ \| \| 38 \| Pregnant Women/ \| \| 39 \| (pregnan* or antenatal or ante-natal or prenatal or pre-natal or perinatal or peri-natal or peripart* or peri-part* or intrapart* or intra-part* or postnatal or post-natal or postpart* or post-part* or maternal).ti,kf. \| \| 40 \| (expectant wom?n or expectant mother? or pregnant wom?n).ti,ab,kf. \| \| 41 \| or/37-40 \| \| 42 \| maternal health services/ or maternal-child health services/ or perinatal care/ or postnatal care/ or prenatal care/ \| \| 43 \| maternal-child nursing/ or midwifery/ \| \| 44 \| health education/ or health promotion/ or patient education as topic/ or prenatal education/ or smoking prevention/ \| \| 45 \| Counseling/ \| \| 46 \| House Calls/ \| \| 47 \| Early Medical Intervention/ \| \| 48 \| Social Support/ \| \| 49 \| (program* or service? or clinic? or intervention?).ti. \| \| 50 \| ((pregnan* or antenatal or ante-natal or prenatal or pre-natal or perinatal or peri-natal or peripart* or peri-part* or intrapart* or intra-part* or postnatal or post-natal or postpart* or post-part* or maternal or midwi*) adj3 (program* or service? or clinic? or intervention?)).ti,ab,kf. \| \| 51 \| ((pregnan* or antenatal or ante-natal or prenatal or pre-natal or perinatal or peri-natal or peripart* or peri-part* or intrapart* or intra-part* or postnatal or post-natal or postpart* or post-part* or maternal) adj3 education*).ti,ab,kf. \| \| 52 \| ((pregnan* or antenatal or ante-natal or prenatal or pre-natal or perinatal or peri-natal or peripart* or peri-part* or intrapart* or intra-part* or postnatal or post-natal or postpart* or post-part* or maternal or midwi*) adj3 (care or healthcare)).ti,kf. \| \| 53 \| ((pregnan* or antenatal or ante-natal or prenatal or pre-natal or perinatal or peri-natal or peripart* or peri-part* or intrapart* or intra-part* or postnatal or post-natal or postpart* or post-part* or maternal or midwi*) adj3 (model? or system?)).ti,ab,kf. \| \| 54 \| ((communit* or neighbo?rhood?) adj3 (program* or service? or intervention?)).ti,ab,kf. \| \| 55 \| ((behav* or risk reduction) adj3 (program* or service? or intervention?)).ti,ab,kf. \| \| 56 \| ((diet* or nutrition* or obes* or weight*) adj3 (program* or service? or intervention?)).ti,ab,kf. \| \| 57 \| ((smok* or tobacco or alcohol or drink* or substance or drug?) adj3 (program* or service? or intervention?)).ti,ab,kf. \| \| 58 \| ((health adj3 (education* or promotion)) or patient education).ti,ab,kf. \| \| 59 \| ((pregnan* or antenatal or ante-natal or prenatal or pre-natal or perinatal or peri-natal or peripart* or peri-part* or intrapart* or intra-part* or postnatal or post-natal or postpart* or post-part* or maternal) adj3 (counsel* or behavi* therap* or cognitive therap*)).ti,ab,kf. \| \| 60 \| or/42-59 \| \| 61 \| fetal mortality/ or infant mortality/ or perinatal mortality/ or maternal mortality/ \| \| 62 \| Pregnancy Outcome/ or Outcome Assessment, Health Care/ or "Outcome and Process Assessment, Health Care"/ \| \| 63 \| exp Pregnancy Complications/ \| \| 64 \| exp infant, low birth weight/ or exp infant, premature/ or Birth Weight/ or Gestational Age/ \| \| 65 \| Breast Feeding/ \| \| 66 \| Depression/pc or Anxiety/pc or Depressive Disorders/pc or Anxiety Disorders/pc \| \| 67 \| health services accessibility/ or access to primary care/ or health equity/ \| \| 68 \| help-seeking behavior/ \| \| 69 \| social inclusion/ \| \| 70 \| exp "Patient Acceptance of Health Care"/ \| \| 71 \| ((preterm* or prematur*) adj3 (birth? or chilbirth? or labo?r or infant?)).ti,ab,kf. \| \| 72 \| (onset adj3 (birth? or chilbirth? or labo?r or infant?)).ti,ab,kf. \| \| 73 \| (miscarriage? or spontanous abortion? or stillbirth? or still birth? or ((fetal or foetal or fetus or foetus) adj (death? or mortality))).ti,ab,kf. \| \| 74 \| (birthweight or birth weight).ti,kf. or (low adj (birthweight or birth weight)).ab. or gestational age.ti,kf. or "small for gestational age".ab. \| \| 75 \| ((pregnancy or maternal or neonat* or newborn? or infant?) adj3 (outcome? or death? or mortality or complication?)).ti,ab,kf. \| \| 76 \| (caesarean? or cesarean? or ((assisted or surg*) adj2 deliver*)).ti,ab,kf. \| \| 77 \| ((intensive care or nicu or icu or itu) adj3 (admission? or admit* or transfer*)).ti,ab,kf. \| \| 78 \| (breastfeeding or breast feeding).ti,ab,kf. \| \| 79 \| (depress* or mood disorder?).ti,ab,kf. \| \| 80 \| (access* adj5 (service? or care or healthcare)).ti,ab,kf. \| \| 81 \| (continuity* adj3 (service? or care or healthcare)).ti,ab,kf. \| \| 82 \| ((engag* or attend*) adj3 (clinic? or service? or program*)).ti,ab,kf. \| \| 83 \| (((health* or care) adj (equit* or inequit* or equalit* or inequalit* or disparit*)) or social inclusion).ti,ab,kf. \| \| 84 \| ((health* or care or help) adj2 seek*).ti,ab,kf. \| \| 85 \| or/61-84 \| \| 86 \| 1 and 41 \| \| 87 \| 36 and 41 and 60 and 85 \| \| 88 \| 86 or 87 \| \| 89 \| afghanistan/ or exp africa/ or albania/ or andorra/ or antarctic regions/ or argentina/ or exp asia, central/ or exp asia, northern/ or exp asia, southeastern/ or exp atlantic islands/ or bahrain/ or bangladesh/ or bhutan/ or bolivia/ or borneo/ or "bosnia and herzegovina"/ or brazil/ or bulgaria/ or exp central america/ or exp china/ or colombia/ or "commonwealth of independent states"/ or croatia/ or "democratic people's republic of korea"/ or ecuador/ or gibraltar/ or guyana/ or exp india/ or indonesia/ or iran/ or iraq/ or jordan/ or kosovo/ or kuwait/ or lebanon/ or liechtenstein/ or macau/ or "macedonia (republic)"/ or exp melanesia/ or moldova/ or monaco/ or mongolia/ or montenegro/ or nepal/ or netherlands antilles/ or new guinea/ or oman/ or pakistan/ or paraguay/ or peru/ or philippines/ or qatar/ or "republic of belarus"/ or romania/ or exp russia/ or saudi arabia/ or serbia/ or sri lanka/ or suriname/ or syria/ or taiwan/ or exp transcaucasia/ or ukraine/ or uruguay/ or united arab emirates/ or exp ussr/ or venezuela/ or yemen/ \| \| 90 \| organisation for economic co-operation and development/ \| \| 91 \| australasia/ or exp australia/ or austria/ or exp baltic states/ or belgium/ or exp canada/ or chile/ or czech republic/ or europe/ or exp france/ or exp germany/ or greece/ or hungary/ or ireland/ or israel/ or exp italy/ or exp japan/ or korea/ or luxembourg/ or mexico/ or netherlands/ or new zealand/ or north america/ or poland/ or portugal/ or exp "republic of korea"/ or exp "scandinavian and nordic countries"/ or slovakia/ or slovenia/ or spain/ or switzerland/ or turkey/ or exp united kingdom/ or exp united states/ \| \| 92 \| european union/ \| \| 93 \| developed countries/ \| \| 94 \| 90 or 91 or 92 or 93 \| \| 95 \| 89 not 94 \| \| 96 \| 88 not 95 \| \| 97 \| limit 96 to yr="2013 -Current" \| \| 98 \| limit 97 to (meta analysis or systematic review or "reviews (maximizes specificity)") \| \| 99 \| (comment or editorial or letter or news).pt. \| \| 100 \| 98 not 99 \| |
| --- | --- | --- | --- | --- | --- | --- | --- | --- | --- | --- | --- | --- | --- | --- | --- | --- | --- | --- | --- | --- | --- | --- | --- | --- | --- | --- | --- | --- | --- | --- | --- | --- | --- | --- | --- | --- | --- | --- | --- | --- | --- | --- | --- | --- | --- | --- | --- | --- | --- | --- | --- | --- | --- | --- | --- | --- | --- | --- | --- | --- | --- | --- | --- | --- | --- | --- | --- | --- | --- | --- | --- | --- | --- | --- | --- | --- | --- | --- | --- | --- | --- | --- | --- | --- | --- | --- | --- | --- | --- | --- | --- | --- | --- | --- | --- | --- | --- | --- | --- | --- | --- | --- | --- | --- | --- | --- | --- | --- | --- | --- | --- | --- | --- | --- | --- | --- | --- | --- | --- | --- | --- | --- | --- | --- | --- | --- | --- | --- | --- | --- | --- | --- | --- | --- | --- | --- | --- | --- | --- | --- | --- | --- | --- | --- | --- | --- | --- | --- | --- | --- | --- | --- | --- | --- | --- | --- | --- | --- | --- | --- | --- | --- | --- | --- | --- | --- | --- | --- | --- | --- | --- | --- | --- | --- | --- | --- | --- | --- | --- | --- | --- | --- | --- | --- | --- | --- | --- | --- | --- | --- | --- | --- | --- | --- | --- | --- | --- | --- | --- | --- | --- | --- |
| Search strategies for all databases are available from the authors on application. |
| Grey Literature: Websites of BirthRights, Birth Companions, Maternity Action, NHSRHO and Sands were also be searched for evidence summaries, alongside the first 150 results of Google Scholar and clinicaltrials.gov. |

**Supplementary Figure 1: Pairwise intersection heatmap showing the number of overlapping primary studies between pairs of reviews**


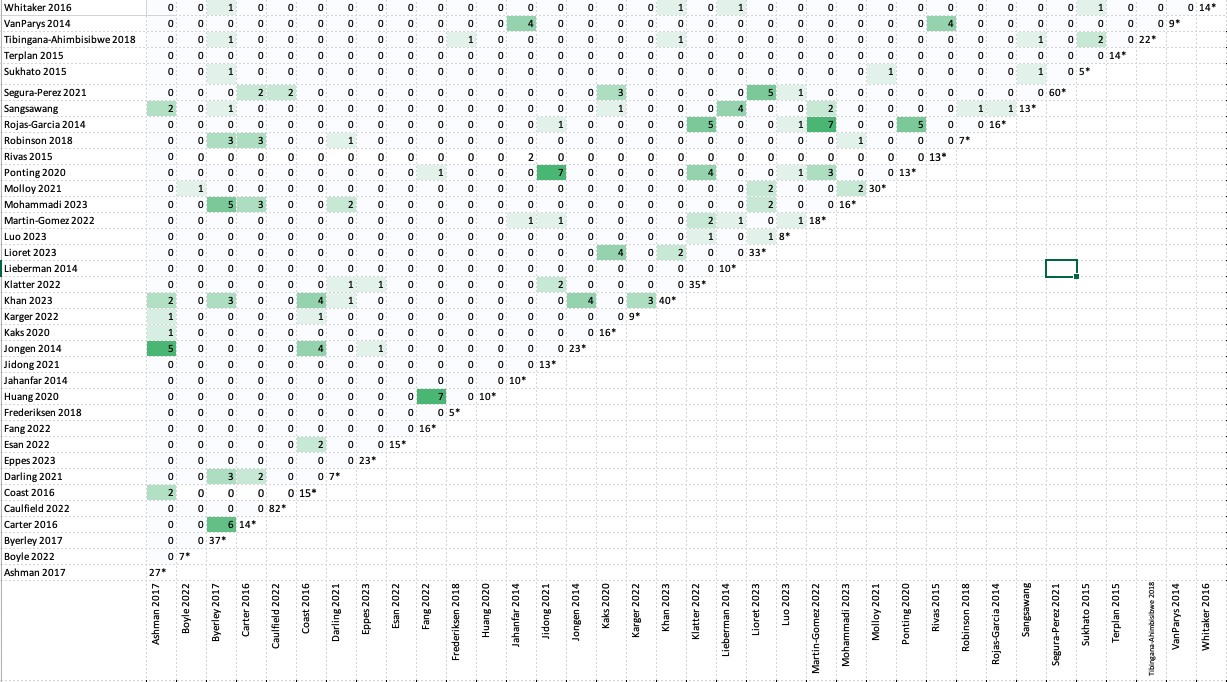


This figure indicates the degree of overlap of primary studies between systematic reviews. The number represents the number of primary studies that are overlapping between the two intersecting reviews. The darker shade of green indicates a greater degree of overlap between the reviews.

**Supplementary Table 3: Summary of included reviews: country and population, timing and context of interventions, level of influence and outcomes reported in relevant primary studies**

| **Authors, date** | **Type of study** | **Countries** | **Search range** | **Number of participants** | **Primary population in studies included in this review** | **Timing of interventions** | **Individual** | **Organisational** | **Community** | **Environment/**  **policy** | **Maternal / Child Health outcome reported (n=significant benefit/assessed)** | **Quality assessment instrument used and appraisal summary** |
| --- | --- | --- | --- | --- | --- | --- | --- | --- | --- | --- | --- | --- |
| Fang 2022 | Meta-analysis - RCT | USA and Canada | 2011-2013 | 131 | Depression | Pregnancy & postnatal | Peer led group or individual support | - | - | - | Depression (n=2/5) | Cochrane collaboration risk of bias tool: 2 at unknown or high risk of bias across majority of domains, 2 at unclear risk for at least one domain |
| Huang 2020 | Meta-analysis-RCT | China, USA, Canada | 2000-2013 | 159 | Postpartum depression | Postnatal | Peer led psychosocial support | - | - | - | Depression (n=2/3) | Cochrane collaboration risk of bias tool: unknown risk of bias across at least one domain |
| Martin-Gomez 2022 | Meta-analysis-RCT | USA | 2007-2013 | 1394 | Socioeconomically disadvantaged | Pregnancy & Postnatal | Psychosocial interventions | - | - | - | Depression | Cochrane collaboration risk of bias tool: 5 high risk, 3 moderate risk, 3 low risk of bias |
| Rivas 2015 | Meta-analysis-RCT | USA, Hong Kong | 2000-2010 | 675 | IPV | Pregnancy & postnatal | Psychosocial interventions | - | - | - | IPV, PTB, LBW, depression, gestational age at birth | Cochrane Handbook for Systematic Reviews of Interventions: 2 low, 1 high risk of bias |
| Rojas-Garcia 2014 | Meta-analysis-RCT | USA, UK, Chile, Turkey | 2001 to 2013 | 1270 | Socioeconomically disadvantaged, majority minoritized ethnic group | Pregnancy & postnatal | Psychosocial interventions | - | - | - | Depression (n=4/10) | EPHPP Quality assessment tool for quantitative studies: All 15 graded moderate or strong quality |
| Sukhato 2015 | Meta-analysis-RCT | USA | 1986 to 2003 | 603 | Adolescents | Pregnancy | Psychosocial interventions | - | - | - | PTB, LBW ( n=0/2) | Risk of bias assessment tool: All studies had low risk of bias in the domains of blinding of participants and personnel, blinding of outcome assessments, selective outcome reporting and other bias. All except one were at low risk of bias from sequence generation. All studies had unclear risk of bias from allocation concealment and two were at high risk for incomplete outcome data. |
| Terplan 2015 | Meta-analysis-RCT | USA | 1995-2012 | 304 | Substance misuse | Pregnancy | Psychosocial interventions | - | - | - | PTB, LBW, neonatal hospital stay, adverse perinatal events | Cochrane collaborations tool for risk of bias: 1 low risk across all domains, 1 high risk in at least one domain, others have at least one domain at unclear risk of bias |
| Whitaker 2017 | Meta-analysis - RCT | USA | 1996-2012 | 3417 | Adolescents | Postnatal | Psychosocial interventions | Interdisciplinary care | - | - | Repeat teen pregnancy | Cochrane collaboration risk of bias tool: for many of the studies, it was difficult to judge the methodological quality because of insufficient reporting of key methodological features. Only one study reported adequate allocation concealment using sealed opaque envelopes. |
| Carter 2016 | Meta-analysis-other study type | USA | 2007-2016 | 927 | low income, ethnic minority, adolescent | Pregnancy | - | Group care | - | - | PTB, breastfeeding, NICU admission | Downs checklist 1998: 4 high, 4 moderate or low quality |
| Mohammadi 2023 | Meta-analysis- other study type | USA, Australia, Spain, France | 1993-2016 | 22,894 | Socioeconomically disadvantaged | Pregnancy | Psychosocial inter | Model of care | - | - | PTB n=6/13; LBW (n=4/12), NICU admissions (n=2/6) or perinatal death (n=0/2) | Cochrane collaboration risk of bias assessment and Newcastle-Ottawa Scale (NOS): One RCT at low risk of bias, all other non-randomised and randomised studies at moderate or high risk of bias |
| Robinson 2018 | Meta-analysis- Other study type | USA | 2004-2016 | 1282 | Low income, ethnic minority, adolescent | Pregnancy & postnatal | - | Group care | - | - | Breastfeeding | John Hopkins Nursing Evidence-Based Practice Rating Scale |
| Ashman 2017 | Narrative | Australia, USA, Canada | 1987-2015 | 56,580 | Women self-reporting as indigenous | Pregnancy & postnatal | Group or individual support and education | Cultural adaptation of care, integrated care models | Community education, community engagement in service development, media campaigns | - | LBW, BF, maternal weight, PTB, dental decay, childhood illness  Community interventions: childhood weight (n=4/5), breast feeding (n=2/4) maternal weight (n=0/1) dental decay (n=0/1)  Culturally adapted interventions: (n=2/3 for BF duration and initiation, PTB (n=3/3) LBW (n=0/3) | The Academy of Nutrition and Dietetics Quality Criteria Checklist for Primary Research: 13 positive, 14 neutral, 0 negative |
| Boyle 2022 | Narrative | Netherlands, USA | 2013-2018 | 937 | Women at high risk or experiencing IPV | Postnatal | Community nurse-led interventions to identify and respond to domestic abuse | - | - | - | IPV (n=2/3) | CCAT critical appraisal tool; scores 88-98% |
| Byerley 2017 | Narrative | Mostly USA | 2001-2017 | Not Reported | Opioid addiction, Adolescents, African Americans, low-income women | Pregnancy | - | Group care | - | - | Neonatal outcomes, attendance (n=1/1), contraception uptake, breastfeeding (n=2/2), rapid repeat pregnancy (n=1/1) | Cochrane collaboration risk of bias assessment and ROBINS-I tool: 10/16 where ROB analysis reported were at low risk of bias across majority of domains |
| Caulfield 2022 | Narrative | USA | 2012-2021 | Not reported | WIC eligible = low-income | Pregnancy & postnatal | - | - | - | WIC: programme administered by US department of agriculture, Food and Nutrition Service providing supplements, education, breastfeeding support and referrals | PTB, LBW, perinatal morbidity and mortality, immunisations, breastfeeding | EPHPP Quality assessment tool for quantitative studies: 28 moderate, 16 weak. |
| Coast 2016 | Narrative | Australia, UK, USA, Israel | 1990-2012 | Not Reported | Ethnic minority | Pregnancy & postnatal | Lay support at home | Integrated, community developed care to overcome cultural barriers | Integrated, community developed care to overcome cultural barriers | - | use, or timing of, antenatal care (n=7/13) or postnatal care (n=1/1) | EPHPP Quality assessment tool for quantitative studies: 4 moderate, remainder weak |
| Darling 2021 | Narrative | USA | 2001-2012 | 3969 | Socially disadvantaged | Pregnancy | Individual counselling | Models of care | - | - | PB, LBW, SGA  PTB n=0/4, LBW n=0.3, SGA. N=1/3 | GRADE: All at least moderate risk of bias |
| Eppes 2023 | Narrative | USA and territories | 2013-2020 | 397 | Socially disadvantaged | Pregnancy & postnatal | Digital interventions | - | - | - | Gestational weight gain, infant weight, breastfeeding, postnatal depression, weight loss | EPHPP Quality assessment tool for quantitative studies: 3 strong, 2 moderate, 1 weak |
| Esan 2022 | Narrative | UK | 1986-2022 | 30,147 | Ethnic Minority | Pregnancy & Postnatal | Professional or peer psychosocial support | Models of Care and cultural adaptation of care | - | Policies for each intervention | LBW, PTB, Depression, perinatal death, BF | MMAT, 2 reported as scoring 4/5, others lower quality |
| Frederiksen 2018 | Narrative | USA | 1987-1992 | 1431 | Adolescents | Pregnancy & postnatal | Individual psychosocial support and education | Interdisciplinary or integrated care | - | - | Repeat teen pregnancy, contraception and immunisation uptake, attendance | USPSTF level of evidence: one level 1 USPSTF- well conducted RCT. Three at Level II USPSTF = controlled but not randomised trials or well-designed cohort or case control studies |
| Jahanfar 2014 | Narrative | USA and Hong Kong | 2000-2011 | 1688 | IPV or socially disadvantaged and at risk of IPV | Pregnancy & postnatal | Home visits, psychosocial interventions, advocacy interventions | - | - | - | IPV, gestational age at birth, LBW, PTB | Cochrane Handbook for Systematic Reviews of Interventions: 1 low risk of bias, others unknown or high risk across majority of domains |
| Jidong 2021 | Narrative | USA | 2008 to 2016 | 681 (not reported in 1 study) | Black African and Caribbean women with depression | Pregnancy & Postnatal | Psychosocial interventions | - | - | - | Depression | Standard Quality Assessment Criteria (QualSyst Tool): All assessed low risk of bias |
| Jongen 2014 | Narrative | Australia | 2000-2011 | 15,398 (not reported in 5 studies) | Indigenous Communities | Pregnancy & postnatal | - | Maternity care adapted or integrated to meet cultural needs | Community engagement in delivery of care | - | antenatal care attendance (n=/09), breastfeeding (n=0/2) and childhood immunization uptake (n=0/2) LBW n=1/13, n=1/4 PTB, 1/3 perinatal mortality | EPHPP Quality assessment tool for quantitative studies: 1 moderate, remainder weak |
| Kaks 2020 | Narrative | UK and USA | 2009-2017 | 6901 | parents in socioeconomic disadvantaged groups | Pregnancy & Postnatal | Peer support home visiting programmes | - | - | - | Breastfeeding (n=2/4, Attendance at care (n=2/2), LBW (n=2/4), PTB (n=1/2), maternal (mental health n=0/3, pregnancy complications n=2/2) | Critical appraisal skills programme (CASP): all articles deemed to satisfy minimum criteria |
| Karger 2022 | Narrative | Australia, Sweden, USA | 2009-2020 | 240,211 | Indigenous and culturally and linguistically diverse infants | Not reported | - | Maternity care adapted or integrated to meet cultural needs | - | expansion of universal healthcare policies | (PTB=3/8, LBW n=1/7 with a further identifying benefit only in medically underserved areas) | Joanna briggs critical appraisal criteria: 5/9 low risk of bias |
| Khan 2023 | Narrative | Chile, Australia, Canada, USA, UK | 1981-2021 | Not reported | Social disadvantage | Pregnancy | - | Models of care, Interdisciplinary care | - | - | Continuity of care: Breastfeeding (n=10/14), Attendance at care (n=13/13), LBW (n=7/14)., PTB (n=12/20), maternal (n=1/1) and neonatal mortality (n=1/4) childhood immunizations (n=0/1)  Tailored midwifery models of care: (PTB (n=6/7), breastfeeding (n=2/2), gestation at first booking appointment or antenatal care coverage (n=6/7) LBW (n=3/6) perinatal mortality (n=0/2)  Integrated care:  infant mortality, (n=1/1), LBW (n=4/5), breastfeeding (n=3/5) and attendance at antenatal care (n=5/6) with mixed effects on PTB (n=3/6) and perinatal mortality (n=1/2) and no reported benefit in uptake of contraception (n=0/1) | Mixed Methods Appraisal Tool: 7 graded 'a' = 100% of the criteria met for that study type |
| Klatter 2022 | Narrative | USA, UK, South Korea, Netherlands, Australia | 2003-2021 | 2441 | Diagnosed Mental Health Disorder | Pregnancy & postnatal | Psychosocial interventions | - | - | - | Depression, PTB | Cochrane risk of bias tool version 2: All at moderate or high risk of bias. |
| Lieberman 2014 | Narrative | USA | 1996-2012 | 865 | Adolescent | Pregnancy & postnatal | Home visits, psychosocial interventions, | - | - | - | Depression | Jadad scale: Out of score 0-5, 5 is highest quality, all scored $\leq$3 so at moderate or high risk of bias |
| Lioret 2023 | Narrative | USA, UK, Netherlands, Australia | 1997-2019 | 6930 | Socioeconomically disadvantaged | Pregnancy & postnatal | Home visits, psychosocial interventions, written or digital education | - | - | - | Breastfeeding practice (n=1/3), birthweight, infant anthropometric measurements | Cochrane risk of bias score version 2: All at moderate risk of bias across at least 1 domain |
| Luo 2023 | Narrative | USA, Japan, Amsterdam | 2011 - 2021 | 768 | Migrant Women | Pregnancy & postnatal | Home visits, psychosocial interventions, community/peer support | - | - | - | Depression (n=1/4) | MMAT: 3 moderate quality (40-60% across 5 domains, 80-100% across 5 domains) |
| Molloy 2021 | Narrative | UK and Netherlands and Germany and USA | 1986-2019 | 5831 | Socioeconomically disadvantaged | Pregnancy & postnatal | Sustained home visits by nurse or social carer | - | - | - | LBW, PTB, postnatal health, common childhood illness, infant mortality, subsequent family planning, breastfeeding, maternal mental health, immunisation compliance, child injuries and maltreatment | NICE quality appraisal checklist for quantitative intervention studies: 6 high quality studies (high quality means >75% of criteria met across 23 items), 17 moderate/high and 1 low quality |
| Ponting 2020 | Narrative | USA | 2006 to 2016 | 1971 | Latina and Black women with depression or anxiety | Pregnancy & postnatal | Psychosocial interventions | - | - | - | Depression (n=3/8 plus 2 showed benefit pre to post treatment) | Cochrane collaboration risk of bias tool: 6 low risk of bias, 5 medium risk of bias, 2 high risk of bias |
| Sangsawang 2019 | Narrative | USA | 2000 to 2015 | 2660 | Adolescent | Pregnancy & postnatal | Psychosocial interventions | - | - | - | Depression (n=0/6) | Quality assessment criteria for evaluating primary research papers from a variety of fields (QUALSYST). 5 fair quality (50-69% assessed domains met), 6 good quality (70-79% met), 2 strong quality (>50% met) |
| Segura-Perez 2021 | Narrative | USA | 2009-2020 | 12,556 | Minoritised ethnic groups | Pregnancy & postnatal | Psychosocial interventions | Group care | - | WIC, baby friendly initiatives | Breastfeeding ((n=10/16) | GRADE: 8 moderate and the rest low or very low risk of bias |
| Tibingana-Ahimbisibwe 2018 | Narrative | Australia, USA, UK, Canada | 1990-2015 | 19,614 | Adolescents | Pregnancy | Psychosocial interventions | Interdisciplinary Care | - | - | PTB, LBW (n=3/4 for both), perinatal death, PNC attendance ((n=2/2) | Effective public health practice project tool: two studies were rated as strong, six as moderate and 13 as weak. |
| VanParys 2014 | Narrative | USA, Australia, Hong Kong | 2004-2011 | 2636 | IPV | Pregnancy & postnatal | Psychosocial interventions | - | - | - | IPV, depression, LBW | Cochrane collaborations tool for risk of bias: 3 at low risk of bias, 3 at unclear risk of bias |
